# Age-Related Reversal of Postural Adjustment Characteristics During Motor Imagery

**DOI:** 10.1037/pag0000120

**Published:** 2016-11-03

**Authors:** Suvobrata Mitra, Nicola Doherty, Hayley Boulton, Elizabeth A. Maylor

**Affiliations:** 1 Division of Psychology, Nottingham Trent University; 2 Department of Psychology, University of Warwick; 3 Division of Psychology, Nottingham Trent University; 4 Department of Psychology, University of Warwick

**Keywords:** aging, posture control, postural sway, motor imagery, dual-tasking

## Abstract

Physical and imagined movements show similar behavioral constraints and neurophysiological activation patterns. An inhibition mechanism is thought to suppress overt movement during motor imagery, but it does not effectively suppress autonomic or postural adjustments. Inhibitory processes and postural stability both deteriorate with age. Thus, older people’s balance is potentially vulnerable to interference from postural adjustments induced by thoughts about past or future actions. Here, young and older adults stood upright and executed or imagined manual reaching movements. Reported arm movement time (MT) of all participants increased with target distance. Older participants reported longer MT than young participants when executing arm movements, but not when imagining them. Older adults’ anteroposterior (AP) and mediolateral (ML) postural sway was higher than young adults’ at baseline, but their AP sway fell below their baseline level during manual imagery. In contrast, young adults’ AP sway increased during imagery relative to their baseline. A similar tendency to reduce sway in the ML direction was also observed in older adults during imagery in a challenging stance. These results suggest that postural response during manual motor imagery reverses direction with age. Motor imagery and action planning are ubiquitous tasks, and older people are likely to spend more time engaged in them. The shift toward restricting body sway during these tasks is akin to a postural threat response, with the potential to interfere with balance during activities of daily living.

Coordination of basic everyday actions such as walking or standing are apparently effortless in the well-functioning adult, but even these highly practiced sensorimotor functions can interfere with a variety of concurrent cognitive tasks, especially in older and balance-impaired individuals (Fraizer & Mitra, 2008; Woollacott & Shumway-Cook, 2002). One interpretation of this interference is that posture control competes with cognitive tasks for shared information-processing resources. For example, a spatial cognitive task may add to demands on limited spatial information processing capacity that is also required for postural control (Maylor & Wing, 1996). An alternative approach stresses psychomotor linkages between cognitive and postural tasks, whereby posture control facilitates the task while also maintaining balance (e.g., when posture control stabilizes the oculomotor system in service of a suprapostural task; Mitra, Knight, & Munn, 2013; Stoffregen, Hove, Bardy, Riley, & Bonnet, 2007).

The present work focuses on the neglected case of a ubiquitous cognitive task that does not mechanically perturb posture control, but functionally links to it, and also places demands on information-processing resources. Activities of daily living are frequently accompanied by thoughts about past, present, or future action sequences (e.g., one might think about aspects of negotiating a flight of stairs and then unlocking the door while approaching with heavy shopping bags). Such motor imagery (MI) tasks not only impose a cognitive load but also activate the motor system in ways that have only recently come to be appreciated. Imagined and physical actions share key behavioral characteristics, such as temporal scaling of movement duration to distance (Papaxanthis, Schieppati, Gentili, & Pozzo, 2002; Sirigu et al., 1996), speed–accuracy trade-off as expressed in Fitts’s law (Decety & Jeannerod, 1995; Stevens, 2005), adherence to biomechanical constraints (Frak, Paulignan, & Jeannerod, 2001; Johnson, 2000), and patterns of actual or simulated effort (Cerritelli, Maruff, Wilson, & Currie, 2000). They also share neurophysiological processes (Bonnet, Decety, Jeannerod, & Requin, 1997; Clark, Tremblay, Ste-Marie, 2004) and cortical activation patterns (de Lange, Hagoort, & Toni, 2005; Grèzes & Decety, 2001; Orr, Lacourse, Cohen, & Cramer, 2008), and their similarities extend beyond cortical processes—imagined movements can modulate corticospinal excitation (Stinear, Byblow, Steyvers, Levin, & Swinnen, 2006), and, in some cases, generate electromyographic (EMG) activity in the involved muscles (Guillot et al., 2007; Lebon, Rouffet, Collet, & Guillot, 2008). Thus, MI incorporates detailed and specific motor planning and also some of the preparatory aspects of motor execution. Suppression of overt movement during MI is thought to be accomplished by a premotor inhibitory mechanism that operates at the brain stem or spinal level (Collet & Guillot, 2009; Jeannerod, 2006), but is incomplete. It does not block autonomic arousal associated with motor planning, for example (Collet, Di Rienzo, El Hoyek, & Guillot, 2013). This inhibition also does not effectively suppress postural adjustments that accompany imagined movement (see de Souza et al., 2015, for a review). As such, MI tasks have significant potential to interact with postural control, especially as the process of aging accumulates deterioration in motor planning (Haaland, Harrington, & Grice, 1993; Trewartha, Endo, Li, & Penhune, 2009), mental imagery (Maylor et al., 2007), and postural control functions (Fraizer & Mitra, 2008).

In our previous work, we asked healthy young adults to stand and imagine reaching movements of the arm, and measured their self-reported movement time (MT) and postural sway (Boulton & Mitra, 2013, 2015). We observed modulation of self-reported MT as a function of stance stability (longer MT in less stable stance), suggesting that parameterization of imagined manual reaching was informed by the current postural context. We also observed modulation of postural sway as a function of imagery task conditions, which showed that postural adjustments were not effectively inhibited during such MI. We followed this up by asking participants to imagine that they were wearing a load on their wrist during the imagined reaching task. This imagined loading of the arm was a purely top-down MI task constraint (i.e., the arm was not in fact loaded during MI), but we still observed postural adjustments in response to this constraint. This indicates that the postural commands that escape inhibition during manual MI are of cortical rather than spinal origin (Boulton & Mitra, 2015).

Here, we focused on the effects of aging on the interaction between manual MI and the control of upright stance. We asked healthy young and older adults to stand in stances of varying baseline stability (open, closed, or semitandem Romberg, in order of decreasing stability), and perform, or imagine performing, reaching arm movements of varying lengths in the anteroposterior (AP) or mediolateral (ML) direction. Reaching from a standing position suspends the arm’s mass away from the body’s main axis. Several postural adjustments might occur in conjunction with the execution of such movement. First, participants might make an anticipatory postural adjustment (APA; Krishnan, Latash, & Aruin, 2012) in the direction opposite to the arm’s movement to counteract its effect on the whole body’s center of mass. Second, participants might use their body sway as a component of the reach (see, e.g., Verheyden et al., 2011), resulting in some body motion in the direction of arm motion. Alternatively, MI might set up an anticipation of postural perturbation that participants counteract by reducing their body sway; if the latter, then the effect ought to be stronger for a less stable stance.

Based on our previous work (Boulton & Mitra, 2013, 2015), we expected postural adjustments to occur even during periods when manual reaching movements were imagined, but not executed. If the adjustments were either APA or body motion in sympathy with (imagined) arm motion, we expected body sway to increase relative to baseline level. If, on the other hand, the predominant postural response was to counteract an expected perturbation, we expected a reduction in body sway relative to baseline, and potentially more so when standing in a less stable stance. Our question of interest was whether there were detectable age-related differences in the type of postural adjustment that occurred during manual MI.

Aging reduces both general postural stability (Rubenstein, 2006) as well as efficiency of voluntary movement planning (Ketcham & Stelmach, 2001), especially in the absence of visual guidance (Haaland et al., 1993). It also negatively affects response planning and the ability to modulate motor plans under high executive control demands (Trewartha et al., 2009). Aging slows mental imagery (Maylor et al., 2007), most likely attributable to working memory deficits (Raz, Briggs, Marks, & Acker, 1999), and it also reduces the effectiveness of inhibitory processes in general (Maylor, Schlaghecken, & Watson, 2005), and in motor control in particular (Schlaghecken, Birak, & Maylor, 2011, 2012). In view of these processes, we predicted that older people might reduce body sway, as though they were minimizing the impact the imagined movement would have had on their balance had it been executed.

Aside from its motoric effects on posture control, MI introduces a cognitive load that might result in dual-task interactions with postural control, especially in older adults (for reviews, see Boisgontier et al., 2013; Fraizer & Mitra, 2008; Woollacott & Shumway-Cook, 2002). Some posture-cognition dual-task studies have shown increased postural sway in older people in particular (e.g., Dault & Frank, 2004; Huxhold, Li, Schmiedek, & Lindenberger, 2006; Maylor, Allison, & Wing, 2001; Maylor & Wing, 1996), but others have reported reduced sway in both older adults and clinical groups (e.g., Andersson, Yardley, & Luxon, 1998; Brown, Sleik, Polych, & Gage, 2002; Deviterne, Gauchard, Jamet, Vançon, & Perrin, 2005; Melzer, Benjuya, & Kaplanski, 2001; Swan, Otani, Loubert, Sheffert, & Dunbar, 2004; Weeks, Forget, Mouchnino, Gravel, & Bourbonnais, 2003). In the present study, if the combined cognitive load of dual-tasking led older participants to prioritize postural control over the MI task, as has been argued for several dual-task settings (Brown et al., 2002; Doumas & Krampe, 2015; Doumas, Smolders, & Krampe, 2008; Rapp, Krampe, & Baltes, 2006), we might also expect to observe a reduction in their postural sway. Unlike in the case of postural adjustments that occur specifically during MI tasks, preferential allocation of time and processing resources to posture control would be a more general means of coping with dual-task demands. In that case, however, we might also expect some negative impact on performance in the MI task (e.g., impaired scaling of imagined MT with distance). Also, we might expect a larger prioritization effect in conditions of lowered baseline stance stability (e.g., in the semitandem Romberg stance).

In contrast to these possibilities in the case of older participants, we expected young participants to exhibit increased body sway relative to their baseline while imagining the manual reaching movements (based on the results of Boulton & Mitra, 2013). For mechanical reasons, we expected both age groups to sway more than their respective baseline levels while executing the reaching movements.

## Method

### Participants

Forty-one young individuals (20 females) from the university community served as the young participants and received £6 ($8) for their participation. Forty-four individuals (27 females) from the local area served as the older participants and received £10 ($13) toward their travel expenses. By self-report, all participants had normal or corrected-to-normal vision, and none had any balance or neurological disorders. Characteristics of the participant pool are summarized in Table 1. It can be seen that the young and older age groups differed as expected in terms of their scores on standardized tests of cognitive functioning, with significantly higher speed but lower vocabulary scores for young than for older participants (e.g., Salthouse, 2010).[Table-anchor tbl1]

The experimental protocol was approved by the University of Warwick’s Humanities and Social Sciences Research Ethics Committee. All participants gave informed consent in writing, and the experimental protocol complied with the code of ethics in the Declaration of Helsinki (World Medical Association, 2013).

### Apparatus and Procedure

**Figure 1 fig1:**
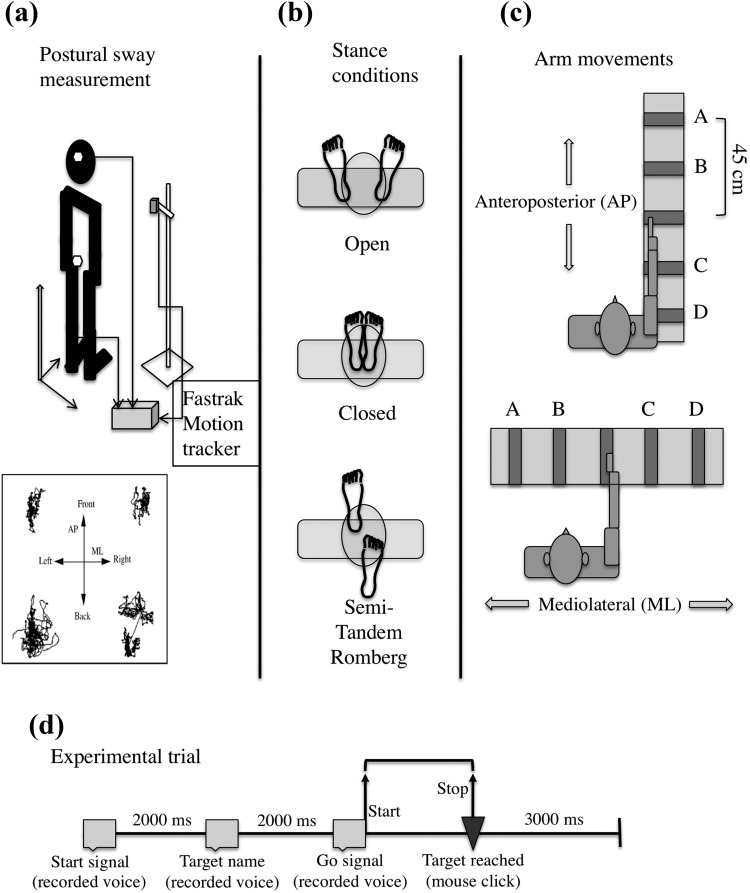
(a) Measurement setup and sample postural sway time series, (b) stance conditions, (c) manual task conditions, and (d) experimental trial construction.

During baseline sway measurement, participants stood barefoot at the designated location marked on the laboratory floor with their arms relaxed by their sides. Polhemus Fastrak motion sensors (Colchester, VT) were attached (using Velcro belts) near the hip (approximately on the lumbar vertebra L5) and on the head (Figure 1a). According to the experimental condition, they stood either in open, closed, or semitandem Romberg stance (Figure 1b). For each of the three stances (open, closed, or semitandem Romberg), participants took up position in the designated location and initially fixated a cross on the laboratory wall at approximately their eye height. Once they felt steady, they were asked to close their eyes and stand quietly for 30 s. During this period, their postural sway data were recorded from the Polhemus sensors. Stance order was randomized.[Fig-anchor fig1]

In the experimental trials, participants were asked to stand barefoot at the designated location marked on the laboratory floor and keep their arms relaxed by their sides. They were also asked to hold a computer mouse in their left hand. Polhemus Fastrak motion sensors were attached to their hip and head, as described in the previous paragraph. Participants were asked to make or imagine reaching movements of their right arm to each of four target areas (1 cm × 35 cm) indicated on a task surface (100 cm × 35 cm). The task surface was positioned at their waist level and was presented in either AP or ML orientation relative to their stance (Figure 1c). The surface was positioned in line with participants’ right shoulder so that the middle target strip, the starting position for each trial, could be reached by raising the lower right arm to an elbow angle just greater than 90**°**.

Each trial (Figure 1d) began with a start signal (a recorded voice saying “Go to the center line”), upon which participants moved (or imagined moving) their right arm to the starting position. Following a 2,000-ms silence, participants heard a recorded voice say the name of the target to be reached (“A,” “B,” “C,” or “D”). Following a further 2,000 ms of silence, they heard the recorded voice say “Go,” upon which they made (or imagined making) the movement to the designated target and clicked the left button of the mouse in their left hand to indicate that they had reached the target. Reaching the target was defined as their index finger entering (and stopping in) the area covered by the target strip. The offset of the go signal set off the timer and participants’ mouse-click (indicating the completion of their movement) stopped it. The next trial began after another 3,000 ms of silence, during which participants returned (or imagined returning) to the arms-by-the-sides standing position. An E-Prime script (Psychology Software Tools, Sharpsburg, PA) controlled the sequencing of trial events, including delivery of the prerecorded auditory instructions, timer functions, and random ordering of movement targets.

The ordering of the three stance conditions (open, closed, and semitandem Romberg) was counterbalanced across participants. A set of arm movements covering each of the four target locations (in random order) comprised a block. There were eight blocks of trials in each of the three stance conditions. First, there were two blocks of physical arm movements with eyes open, followed by two blocks of imagined movements with eyes closed (which served as imagery practice). These were followed by four blocks of experimental trials in which participants stood with their eyes closed and imagined the designated arm movements. Participants were rested for 5 min between the three stance conditions.

Participants’ instructions for the arm movements were to simply move (or imagine moving) their index finger to the named target as swiftly as possible without sacrificing accuracy. In the case of imagery trials, participants were asked to stand quietly, and the instructions made it clear that it was important not to actually make any arm or head movements. Thus, there was no scope to mime the actions being imagined. No explicit reference was made in the instructions to specific imagery modalities (e.g., visual or kinesthetic), but the instructions stressed that participants should imagine *making* the movements. As the reaching task used in this study is commonly a visuomotor task in daily life, it would be confusing for participants if an explicit contrast was made between kinesthetic and visual imagery, and participants were asked to desist from the latter. Thus, we used the emphasis on imagining making the movements, along with the physical movement experience preceding imagery, to stress the kinesthetic perspective.

MT was measured on a per-movement basis. Postural sway was measured on a per-block basis, such that each sway time series contained body sway during four arm movements made (or imagined) while standing in a particular stance (open, closed, or semitandem Romberg). As in our previous work (Boulton & Mitra, 2013, 2015), per-block sway measurement was used to capture postural effects of imagining a sequence of manual actions, as would be the case during activities of daily living. Finally, half of the participants performed the arm movements in the ML direction and the other half in the AP direction. We included the direction of imagined movement as a between-subjects factor to prevent sporadic carry-over effects in imagery that were reported by some pilot participants (as in Mitra et al., 2013). During MI in the second task orientation, they reported interference from imaging the task in the first orientation. Making task orientation a between-subjects factor ensured that each participant only ever encountered the task setup in a single orientation.

### Measures, Design, and Data Analysis

We measured self-reported MT as the interval between the “Go” signal and the participants’ mouse-button press indicating completion of their physical or imagined arm movement. We analyzed MT using a 2 (age: young, older) × 3 (stance: open, closed, semitandem Romberg) × 4 (arm movement target: A, B, C, D) × 2 (arm movement direction: ML, AP) × 2 (task: physical arm movement, imagined arm movement) mixed ANOVA, with all except age and arm movement direction as within-subjects factors. In the physical trials, there were five occasions in which the participant failed to click the mouse button to indicate the end of arm movements they executed (one case in young, and four cases in older participants). Mean substitution was used in these cases. In the imagery trials, two young and four older participants recorded several cases of self-reported MT greater than 2.5 standard deviations from the group mean. As it could not be ascertained whether these constituted a failure to perform the imagery task or to indicate the end of imagined movements in a timely manner, these participants’ data were removed from all analyses.

We recorded participants’ AP and ML postural sway from hip-attached Polhemus sensors at 60 Hz (with a static accuracy of 0.012 cm root mean square [RMS] with 4-ms latency; Figure 1a). The postural sway time series observed in the human upright stance are nonstationary (Carroll & Freedman, 1993; Riley, Balasubramaniam, & Turvey, 1999) in that they contain both local fluctuations of position as well as drift of mean position over time. Zatsiorsky and Duarte (2000) term these the “trembling” and “rambling” aspects of postural sway. Consider the four examples of postural sway time series shown in the inset at the bottom of Figure 1a. The top-left example shows local sway fluctuations (“tremble”) overlaid with slower, mostly front–back drifts of position (“ramble”). The bottom left example shows a similar level of tremble, but an increased level of ramble (including several long excursions to the right of the most commonly occupied region). In the bottom-right example, local fluctuations appear to have occurred in one region for one part of the trial, and in the second region in the other part, with a single, longer time-scale positional drift linking the two regions. This nonstationarity means that a gross estimate of variability such as the standard deviation of body position taken over extended time (e.g., over the full course of these example time series) would be affected by both short time-scale postural jitter as well as longer time-scale position drift. In other words, a single gross measure of variability is not effective when there is variability of interest at different time-scales (i.e., short time-scale fluctuations and longer time-scale positional drift). An established technique for isolating the variability associated with a characteristic time-scale in time series data is to use moving window standard deviation (see, e.g., McNevin & Wulf, 2002; Mitra, 2003). As the time window over which variability is calculated (e.g., of 1-s duration) moves along the time series, the average variability is less influenced by longer time-scale positional drift, and gives a more accurate estimate of the nature of the dynamics at that particular time-scale. The key issue in the use of moving window statistics is how to determine the characteristic time-scales in postural sway data. One approach is based on the observation (Collins & De Luca, 1993, 1995) that over shorter time windows (<1 s), sway data show the property of persistence (i.e., there is an average tendency to continue motion in the current direction, giving an overall positive correlation between past and future movements). Over longer time windows (>1 s), the sway data are antipersistent (i.e., have the tendency to reverse direction, yielding an overall negative correlation between past and future motions). It has been suggested that this temporal structure composed of shorter time-scale tremble and longer time-scale ramble might correspond to the two key components of the postural control during unperturbed upright stance—exploratory (open-loop) movements over shorter time-scales to gather information about the state of the postural system, and performatory (closed-loop) motions over longer time-scales to confine body position within safe bounds (Mitra, Balasubramaniam, Riley, & Turvey, 1996; Riley, Mitra, Stoffregen, & Turvey, 1997; Riley, Wong, Mitra, & Turvey, 1997).

As in our previous studies (Boulton & Mitra, 2013, 2015; Mitra, 2003), we used two measures of postural sway to estimate the two characteristic time-scales discussed above. We estimated short time-scale (STS) sway along the AP and ML directions as the average moving window standard deviation of position within all nonoverlapping time windows of 1 s. Thus, STS sway provided an estimate of the frequency and amplitude of postural adjustments at time-scales shorter than 1 s. We analyzed long time-scale (LTS) sway as the RMS drift of body position across all windows of 1-s duration in the time series. Thus, a sway time series containing higher frequency or amplitude of microadjustments would yield a greater STS sway magnitude, whereas the LTS sway level would depend more on the absolute distance traversed by body position. The two measures covary, but in varying amounts, as when there is higher frequency of responding but position is confined to a smaller area, or when there are weaker or infrequent adjustments while position drifts over a wider area.

We analyzed participants’ AP and ML sway using a 2 (age: young, older) × 3 (stance: open, closed, semitandem Romberg) × 2 (arm movement direction: ML, AP) × 3 (task: baseline, imagined arm movement, physical arm movement) mixed ANOVA with stance and task as within-subjects factors and age and arm movement direction as between-subjects factors. In all analyses of variance, the significance level for omnibus effects was set to *p* < .05. A Bonferroni correction was applied (.05/*n*; *n* = number of contrasts) to post hoc mean comparisons. As already noted, the physical movement condition always preceded the MI condition, and unlike the MI and baseline sway conditions, it was carried out with eyes open. Our hypotheses focused on differences between baseline and MI conditions, not between MI and physical movement. As such, we did not interpret the latter.

## Results

### Overview of Age-Related Effects

**Figure 2 fig2:**
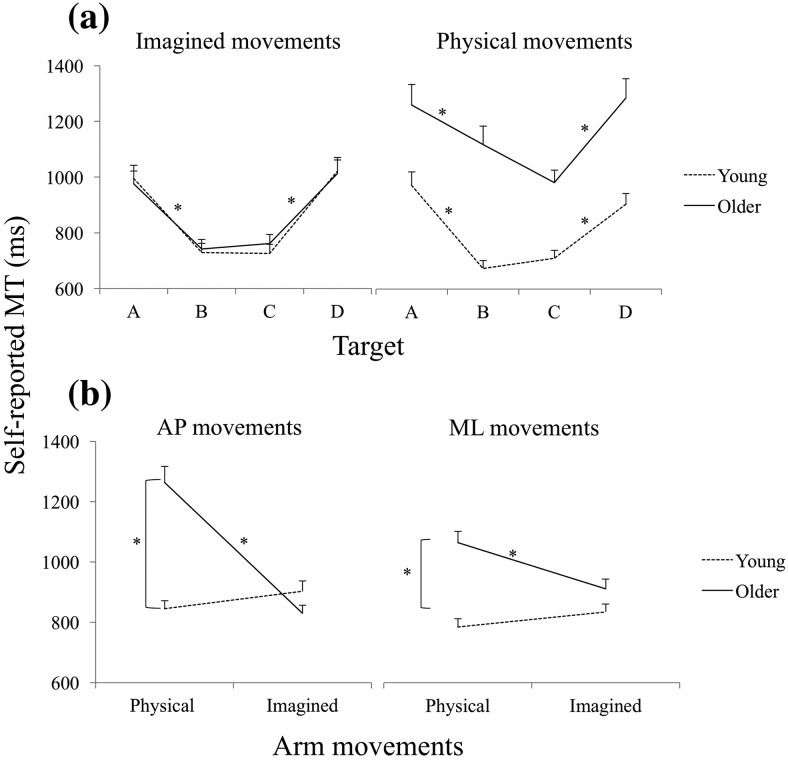
(a) Self-reported movement times (MTs) of young and older participants to different targets. (b) Self-reported MT of physical and imagined arm movements made in the anteroposterior (AP) and mediolateral (ML) directions. Error bars indicate standard error. * Indicates a statistically significant difference.

**Figure 3 fig3:**
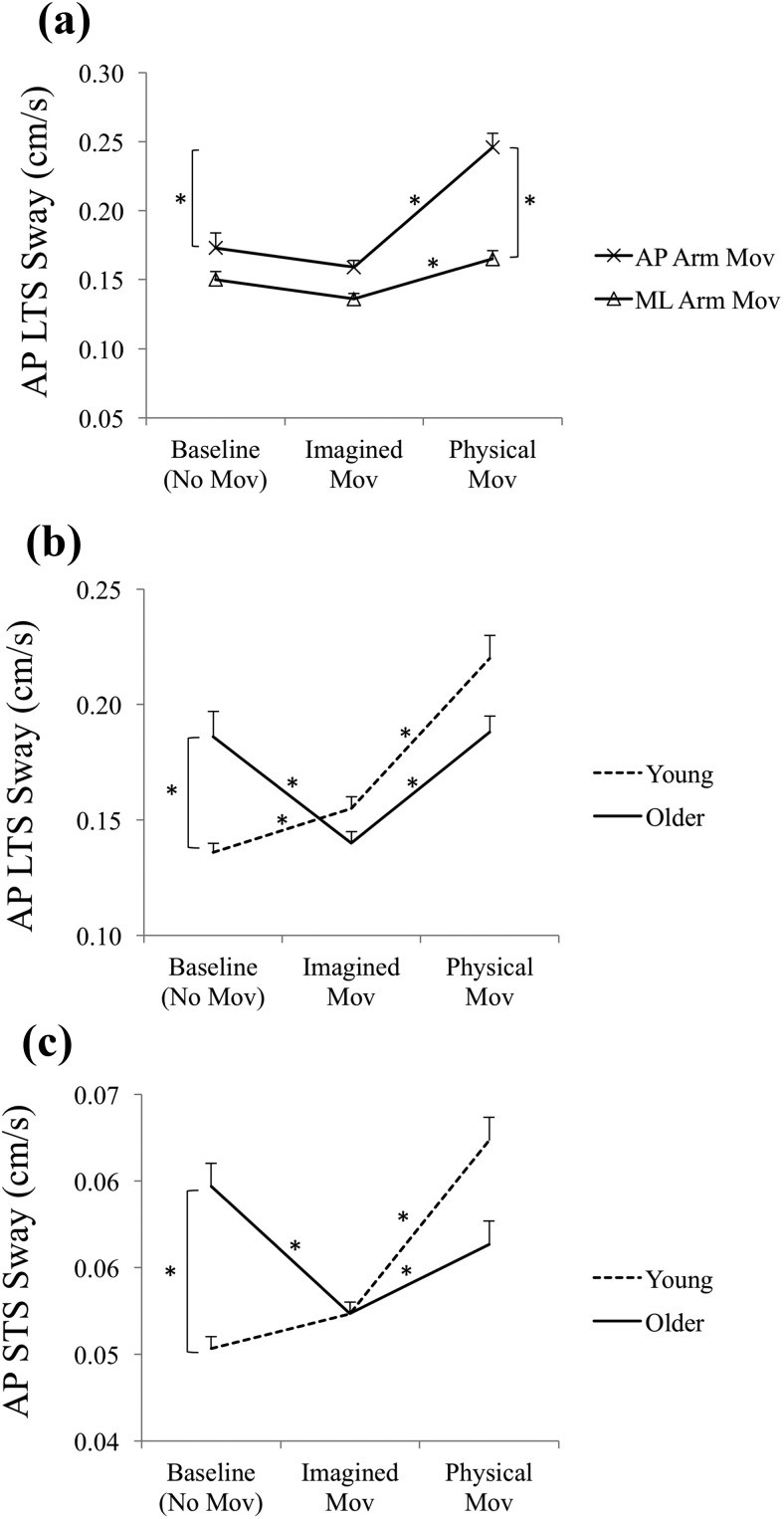
(a) Anteroposterior (AP) long time-scale (LTS) sway during arm movements in AP and mediolateral (ML) directions during baseline (no arm movement task), imagined, and physical arm movements. (b) AP LTS sway of young and older participants during baseline, imagined, and physical arm movements. (c) AP short time-scale (STS) sway of young and older participants during baseline, imagined, and physical arm movements. Error bars indicate standard error. * Indicates a statistically significant difference.

**Figure 4 fig4:**
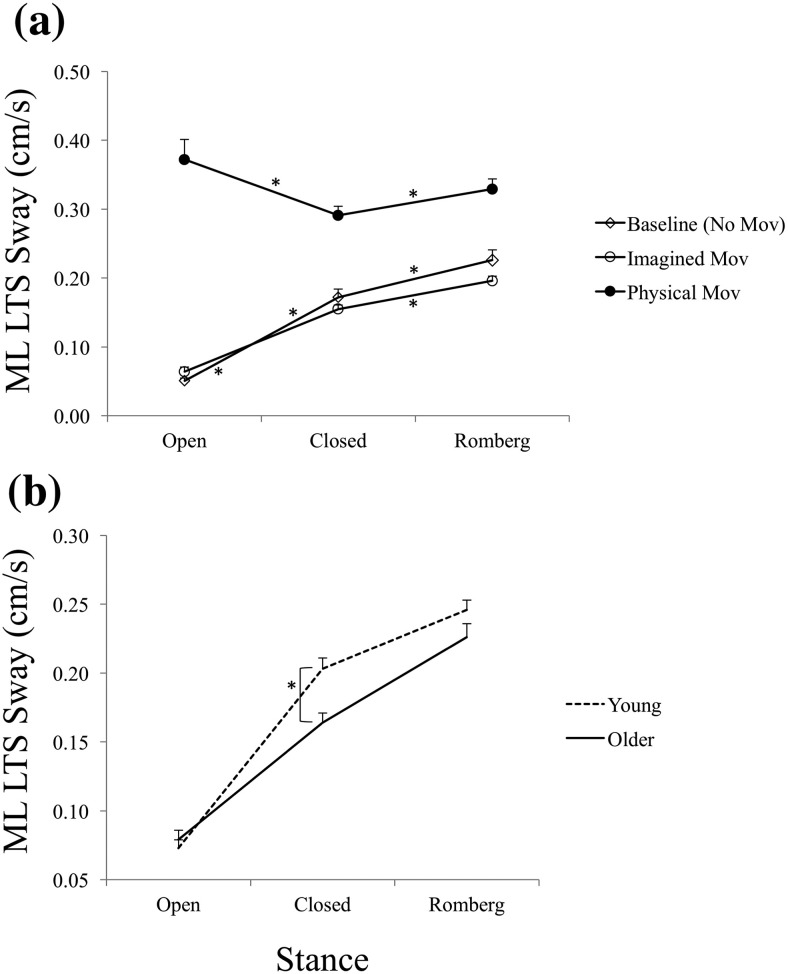
(a) Mediolateral (ML) long time-scale (LTS) sway in open, closed, and semitandem Romberg stances during baseline, imagined, and physical arm movement conditions. (b) ML LTS sway of young and older participants during imagined movements. Error bars indicate standard error. * Indicates a statistically significant difference.

Analysis of self-reported MT data showed that both young and older participants scaled MT to distance as expected. Older participants reported slower physical movements, but their imagined MTs were nearly identical to those of young participants (Figure 2a). Older participants’ AP sway decreased during MI relative to their baseline, whereas young participants’ AP sway increased during imagery (Figures 3b and 3c). Older participants’ ML sway was lower than young participants’ only in the closed stance (Figure 4b). Even though baseline ML sway was lowest in the open and highest in the semitandem Romberg stance for both age groups (Figure 4a), both groups also swayed least in closed stance when they physically made the arm movements (Figure 4a). This suggests that the closed stance, which had the smallest support surface area, was felt to be the most challenging stance in the context of arm movements.[Fig-anchor fig2][Fig-anchor fig3][Fig-anchor fig4]

### Self-Reported MT

The main effect of target was significant, *F*(3, 225) = 61.51, *p* < .0001, η_p_^2^ = .45; movements to farther targets, A and D, took longer (Figure 2a). The main effect of task was significant, *F*(1, 75) = 11.48, *p* < .01, η_p_^2^ = .13 (MT for physical movements was longer than for imagined movements). The main effect of age was significant, *F*(1, 75) = 5.05, *p* < .05, η_p_^2^ = .06 (older participants reported longer MT), as was the interaction between age and task, *F*(1, 75) = 24.06, *p* < .0001, η_p_^2^ = .24; older participants’ MT was longer in physical than in imagined movements; young participants’ MT did not differ between task conditions, nor from older participants’ MT in the imagined movement condition; and older participants’ MT was significantly longer than young participants’ in the case of physical movements (Figure 2a). The interaction between age, task, and arm movement direction was also significant, *F*(1, 75) = 4.14, *p* < .05, η_p_^2^ = .05; the difference between older participants’ MT in physical and imagined movements was greater for AP than ML movements (Figure 2b). The interaction between age, task, arm movement direction, and target was also significant, *F*(3, 225) = 3.63, *p* < .05, η_p_^2^ = .05; the interaction between age, task, and arm movement direction shown in Figure 2b was more pronounced for the farther targets.

### AP Postural Sway

#### LTS sway

The main effect of arm movement direction was significant, *F*(1, 75) = 19.78, *p* < .0001, η_p_^2^ = .21 (AP LTS sway was greater for AP than ML arm movements). The main effect of task was significant, *F*(2, 150) = 27.66, *p* < .0001, η_p_^2^ = .27 (AP LTS sway was greater during physical movements than during imagined-movement or no-movement-baseline conditions; it did not differ in the latter two conditions). The interaction between task and stance was significant, *F*(4, 300) = 83.79, *p* < .01, η_p_^2^ = .05; AP LTS sway in semitandem Romberg stance was greater than in open or closed stance only in the physical movement condition. The interaction between task and arm movement direction was significant, *F*(2, 150) = 8.28, *p* < .001, η_p_^2^ = .10 (Figure 3a). For AP arm movements, AP LTS sway was significantly greater during the physical than imagined- or no-movement condition. The pattern was identical for ML arm movements, except that the difference was numerically smaller. Also, AP LTS sway was greater for AP than ML physical arm movements, but this difference was not significant in the imagined or no-movement-baseline conditions. The interaction between task and age was also significant, *F*(2, 150) = 14.24, *p* < .0001, η_p_^2^ = .16 (Figure 3b). Young participants’ AP LTS sway increased significantly from baseline to imagined arm movements, and from imagined to physical arm movements. Older participants’ AP LTS sway was greater than young participants’ in the baseline condition, but dropped significantly during imagined arm movements. Their AP LTS sway was greater during physical than imagined arm movements, but did not differ between baseline and physical movements.

#### STS sway

**Figure 5 fig5:**
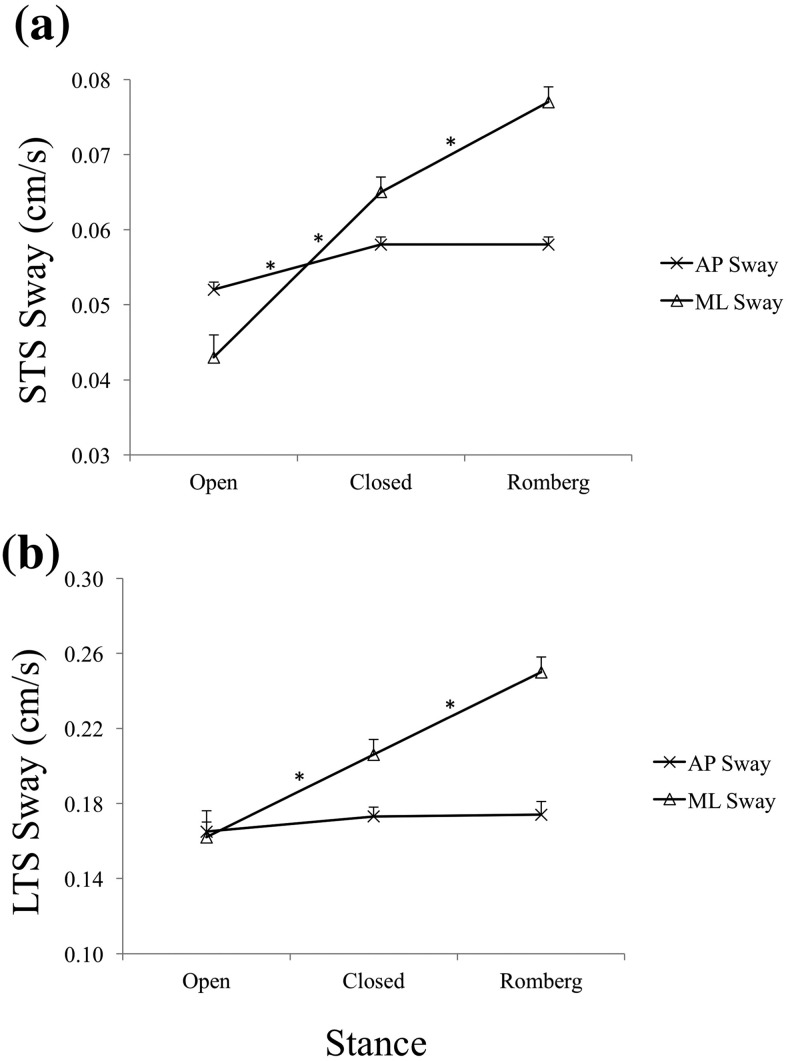
(a) Effect of stance on anteroposterior (AP) and mediolateral (ML) short time-scale (STS) sway. (b) Effect of stance on AP and ML long time-scale (LTS) sway. Error bars indicate standard error. * Indicates a statistically significant difference.

The main effect of arm movement direction was significant, *F*(1, 75) = 16.36, *p* < .0001, η_p_^2^ = .18 (AP STS sway was greater for AP than ML arm movements). The main effect of task was significant, *F*(2, 150) = 13.72, *p* < .0001, η_p_^2^ = .15 (AP STS sway was greater during physical than imagined movements; baseline sway did not differ significantly from imagined or physical movement conditions). Unlike in the case of AP LTS sway (Figure 5b), the main effect of stance was significant, *F*(2, 150) = 11.99, *p* < .0001, η_p_^2^ = .14 (Figure 5a); AP STS sway in open stance was lower than in closed or Romberg stance. The interactions between task and movement direction, *F*(2, 150) = 15.92, *p* < .0001, η_p_^2^ = .18, and between task and age, *F*(2, 150) = 16.03, *p* < .0001, η_p_^2^ = .18 (Figure 3c), were also significant and had the same pattern as previously reported for AP LTS sway.[Fig-anchor fig5]

### ML Postural Sway

#### LTS sway

The main effect of arm movement direction was significant, *F*(1, 75) = 15.69, *p* < .001, η_p_^2^ = .17 (ML LTS sway was greater for AP than ML arm movements). The main effect of task was significant, *F*(2, 150) = 136.80, *p* < .0001, η_p_^2^ = .65 (ML LTS sway was greater during physical movements than during imagined-movement or no-movement-baseline conditions; it did not differ in the latter two conditions). The main effect of stance was significant, *F*(2, 150) = 46.04, *p* < .0001, η_p_^2^ = .38; ML LTS sway increased significantly from open to closed, and from closed to Romberg stances (Figure 5b). The interaction between task and movement direction was significant, *F*(2, 150) = 8.99, *p* < .001, η_p_^2^ = .11; the pattern was exactly as in the case of AP LTS sway (Figure 3a). The interaction between task and stance was significant, *F*(4, 300) = 38.55, *p* < .0001, η_p_^2^ = .34; ML LTS sway increased from open to closed to Romberg stance in the baseline and imagined-movement conditions, but the pattern differed during physical arm movements (Figure 4a)—ML LTS sway in this case was less in closed than in open or Romberg stances, and did not differ in the latter two stances. The interaction between task, stance, and movement direction was also significant, *F*(4, 300) = 7.01, *p* < .0001, η_p_^2^ = .09. The pattern shown in Figure 4a was the same in both movement directions, but ML LTS sway was greater for AP than ML movements in the physical condition.

There were no significant aging effects on ML LTS sway in this overall analysis, but in contrast to the baseline and imagined-movement conditions, physical movement showed a reduction in ML LTS sway in closed relative to open stance (Figure 4a), suggesting that participants particularly restricted ML LTS sway when performing arm movements while in closed stance. To explore whether young and older participants’ sway may have differed, particularly in closed stance, when they imagined rather than performed the movements, we analyzed ML LTS sway in the imagined-movement condition only using a 2 (age) × 3 (stance) × 2 (arm movement direction) mixed ANOVA. The main effect of stance was again significant, *F*(2, 170) = 227.10, *p* < .0001, η_p_^2^ = .73; ML LTS sway differed between all three stances, with the least sway in the open stance and the most in the semitandem Romberg stance. Additionally, the interaction between stance and age was now also significant, *F*(2, 170) = 4.84, *p* < .01, η_p_^2^ = .05. Older participants swayed less than young participants when imagining arm movements specifically while standing in the closed stance (Figure 4b).

#### STS sway

The main effects of arm movement direction, *F*(1, 75) = 10.06, *p* < .01, η_p_^2^ = .12, task, *F*(2, 150) = 90.00, *p* < .0001, η_*p*_^*2*^ = .55, and stance, *F*(2, 150) = 204.38, *p* < .0001, η_p_^2^ = .73 (Figure 5a), were significant, as were the interactions between task and movement direction, *F*(2, 150) = 12.03, *p* < .0001, η_p_^2^ = .14, task and stance, *F*(4, 300) = 61.82, *p* < .0001, η_p_^2^ = .45, and task, stance, and movement direction, *F*(4, 300) = 5.343, *p* < .001, η_p_^2^ = .07. In all cases, the patterns were identical to those reported for ML LTS sway. We also conducted the ANOVA separately for the imagined-movement condition, but unlike in the case of ML LTS sway, the interaction between stance and age was not significant on ML STS sway.

## Discussion

As in our previous studies (Boulton & Mitra, 2013, 2015), participants scaled their self-reported MT to target distance similarly in physical and imagined arm movements, confirming that they performed the task in the expected manner in both cases. Between-subjects variability of self-reported MT was low for both physical and imagined movements, and for both young and older participants (see Figure 2), suggesting that distance-scaling performance was consistent across participants. In the case of physical movements (Figure 2a, right panel), older participants were slower than young participants, as expected, but this difference did not appear in imagined movements (Figure 2a, left panel). As the imagined MTs reported by both groups were similar to young participants’ physical MTs, one interpretation is that older people failed to reflect their motor slowing in trajectory planning during imagery. This could be due to age-related deterioration in the coupling between task-level action planning and effector-level movement control (Saltzman & Kelso, 1987; Wolpert & Kawato, 1998), which may reduce correspondence between the motor plan established during imagery and the delivery of all its aspects in execution (e.g., Skoura, Papaxanthis, Vinter, & Pozzo, 2005). The absence of an effect of age in the case of imagined movements suggests, at least, that older participants were not aware of planning faster movements than they would execute under those conditions.

Note that patterns of age-related loss of correspondence between overt and covert performance have also been observed in domains other than pointing arm movements. In the contrast between overt and covert articulation of speech (i.e., vocal and subvocal speech), overt articulation rates are slower in older adults (e.g., Multhaup, Balota, & Cowan, 1996; Smith, Wasowicz, & Preston, 1987), but covert articulation rates are not significantly different between older and young adults (e.g., Maylor & Wing, 1996; Watson, Maylor, & Bruce, 2005). In the case of nonpointing arm movements, older adults do not retain the level and consistency of temporal similarities between overt and covert arm movements observed in young adults (Skoura, Personnier, Vinter, Pozzo, & Papaxanthis, 2008). In particular, older adults show deficiencies in integrating inertial properties of the arm into their action representation during covert movements (e.g., Personnier, Paizis, Ballay, & Papaxanthis, 2008). In the case of sit-to-stand movements (timed up-and-go), older people report faster times during MI relative to execution (Bridenbaugh et al., 2013; but see Skoura et al., 2005). In the case of walking, older people’s MT during MI fails to increase with their execution time over longer distances (>20 m; Schott & Munzert, 2007), but under conditions of spatial constraint (e.g., narrow walkway), older people can overestimate walking time during MI relative to execution time (Personnier, Kubicki, Laroche, & Papaxanthis, 2010). These and the present results all point to an age-related loss of timing correspondence between the feedforward aspect of motor planning that is captured in MI, and the combination of feedforward and feedback processes that occur during physical movements.

Older and young participants showed clear differences in their AP LTS and STS sway patterns across the task conditions (Figures 3b and 3c). As expected, older participants swayed more than the young in the baseline condition. Whereas young participants’ AP LTS sway was greater (and STS sway marginally so) during imagery compared with baseline, older participants’ AP LTS and STS sway were significantly reduced during imagery compared with baseline. Young participants seem to have prioritized postural facilitation of the planned arm movement, whether by planning an APA to compensate for the shift in the body’s center of mass or by using body sway as a component of the reaching movement. Like our previous studies in this series (Boulton & Mitra, 2013, 2015), this study was concerned with the general pattern of postural adjustments accompanying a sequence of imagined movements, as would be common in everyday settings, and so collected postural sway data across blocks of arm movements. Thus, the postural effects here encompassed periods of carrying out MI as well as periods of anticipating or recovering from MI. Further studies using per-movement body motion and EMG recording could ascertain how sway modulation is distributed immediately before, during, or immediately after each component movement of an MI sequence. As MI during daily activities is likely to be of sequences of movements, and occur over periods of locomotion or maintenance of stance, modifications of postural adjustment during these periods are of interest regardless of their exact phasing during those periods.

In contrast, older participants appear to have undertaken a restriction in body sway during MI. One possibility is that this was a bracing action against an expected postural destabilization due to the planned arm movement. In their own ways, both age groups failed to inhibit a postural adjustment when the planned movement was imagined but not executed. In the case of physical movements, young participants expectedly swayed more compared with baseline (whether to compensate for the shift in center of mass or to use trunk motion as a component of the reaching movement). Contrary to expectation, neither measure of older participants’ body sway during physical arm movement increased relative to their baseline. This pattern, combined with its analogue in the MI condition, could be an indication of older adults’ general tendency to restrict body sway during not just imagined but also physical arm movements. However, as the physical movements were performed with eyes open, whereas the baseline and MI conditions were conducted without vision, it is possible that the physical movement condition affected young and older participants differently. It is well known that the contribution of visual information to the control of upright stance increases with age (Matheson, Darlington, & Smith, 1999; Perrin, Jeandel, Perrin, & Béné, 1997; Poulain & Giraudet, 2008; Teasdale, Stelmach, & Breunig, 1991). The absence of an increase in older participants’ sway between the baseline and physical movement conditions could be at least partly due to the possibility of using vision to stabilize stance in the latter condition. In any case, it was the difference between young and older adults’ sway during MI relative to baseline (under identical conditions) that was the key contrast of interest here.

The stance manipulation in this study was designed to create different levels of postural challenge to see whether any age-related differences in postural adjustments during MI were affected by the level of postural threat in the task situation. The pattern of baseline sway in the three stances showed that, in the AP direction, open stance had less sway than the other two stances (which did not differ from each other) on both measures of sway (see Figure 5). The stance stability manipulation was therefore partially successful in AP, but the age effect on both measures of AP sway during MI did not differ between stances. Thus, the reversal of postural adjustment between young and older participants in the AP direction appeared to occur irrespective of stance stability.

The stance difficulty manipulation had a clearer effect in the ML direction, as baseline sway increased from open to closed to semitandem Romberg stance (Figure 4a). The semitandem Romberg stance had the least ML stability according to the level of sway recorded in quiet stance (Figure 4a), so we might have expected the highest level of attempted sway restriction in this stance. However, participants actually restricted their ML sway (on both measures) the most in closed stance when they physically performed arm movements (Figure 4a). In line with this, in the MI condition, older participants reduced their ML LTS sway relative to young participants when imagining arm movements in closed stance (Figure 4b). As the closed stance offered the smallest overall support surface area of the three stances, participants may have felt the greatest need to brace against perturbation (during movement execution) while in this stance. The fact that older participants showed less ML LTS sway in this stance than young participants also when imagining arm movements suggests that they expected, and adjusted for, a greater perturbation to their postural stability in this stance. Note that the age effect in the MI condition occurred only on the LTS measure of ML sway. Thus, older adults’ ML sway restriction focused on limiting longer time-scale drift of body position. This pattern can be seen in the context of previous work on the ramble and tremble decomposition of postural sway showing that the longer time-scale ramble aspect is more accessible to volitional control, and is therefore more readily reduced in response to task demands (Danna-Dos-Santos, Degani, Zatsiorsky, & Latash, 2008).

As noted in the introduction, an alternative interpretation of older participants’ sway reduction might be that, faced with the cognitive load of concurrently controlling stance and imagining or executing arm movements, they prioritized posture control (Brown et al., 2002; Doumas & Krampe, 2015; Doumas et al., 2008; Rapp et al., 2006), resulting in reduced sway. Thus, rather than being the result of a specific failure to inhibit postural adjustments triggered by MI, reduced sway in older participants was the result of a general strategy for coping with the pressure of dual-tasking by preferentially allocating limited cognitive resources to posture control. If so, we might have expected a concomitant deterioration in older participants’ performance in the MI task, and more so as the difficulty of the balancing task increased from open to closed to semitandem Romberg stance. However, timing variability during MI was very similar in both groups, as was the rate of increase in MT with increasing target distance (Figure 2a). Also, there was no effect of the stance stability manipulation on MI task performance. It could be argued, however, that the shorter MTs reported by older participants during MI were due to the allocation of less time or processing resources, but there were no other indicators of MI performance decrement (e.g., deterioration in scaling) to support that possibility.

A clear performance tradeoff between posture control and MI would have strongly suggested prioritization of limited cognitive resources, but its absence does not negate this possibility as posture-cognition dual-tasking experiments rarely set up a zero-sum scenario in this respect. Unquantifiable cognitive effort during baseline measurement, changes to cognitive focus on postural sway, spare cognitive capacity, or even the level of arousal may all mitigate against a direct performance tradeoff (Fraizer & Mitra, 2008). The present pattern of increased sway in young, but decreased sway in older, adults is unusual, however, and a simple explanation would be that young adults planned postural motions that would have facilitated the imagined arm movement, whereas older adults reduced sway to brace against the potential perturbation.

Manual MI does not mechanically perturb the standing body, and if it is a purely cognitive operation, should not present any mechanical demands beyond those associated with quiet standing. The observed differences in body sway between young and older participants during manual MI extend and elaborate previous work showing that sway restriction during manual MI can be induced in young adults by introducing a top-down task constraint such as an imagined load on the arm, but only when the postural task is sufficiently challenging (Boulton & Mitra, 2015). A likely reason why older participants restrict postural sway relative to quiet standing could be that they brace the body against the perturbation implied by the planned movement. Another possibility could be that they act strategically to stabilize the body as a platform for the planned arm movement. In either case, the motor commands in question are not effectively inhibited in the absence of movement execution. As such, the process could be viewed as a particular type of postural prioritization that occurs during MI tasks, particularly in older people.

Taken together, these results suggest that aging introduces a postural threat response into the process of planning manual movements. Just the thought of manual actions acquires the potential to interfere with postural support for ongoing sensorimotor coordinations. This change occurs while the efficiency of motor planning and modulation also declines (Haaland et al., 1993; Ketcham & Stelmach, 2001; Trewartha et al., 2009), as does working memory capacity, which makes imagery less efficient (Maylor et al., 2007; Raz et al., 1999). As a result, older people are also more likely to spend longer periods of time engaged in motor planning. Further work examining the impact of MI on walking and other frequently performed activities of daily living would therefore be of significant benefit in understanding the factors that reduce psychomotor confidence and mitigate against active, independent living in old age.

## Figures and Tables

**Table 1 tbl1:** Participant Background Details

Variable	Young	Older
*N* (M/F)^a^	41 (21/20)	44 (17/27)
Age range	18–30	65–80
Mean age in years (*SD*)	20.7 (2.4)	70.9 (4.1)
Mean height in m (*SD*)	1.72 (0.10)	1.63 (0.10)
Mean weight in kg (*SD*)	65.1 (10.8)	71.5 (11.5)
Speed (*SD*)^b^	73.4 (9.9)	51.0 (7.2)^e^
Vocabulary (*SD*)^c^	17.8 (3.4)	25.0 (4.3)^e^
Digit span (*SD*)^d^	15.6 (3.9)	16.4 (3.4)
^a^ Number of participants (males/females). ^b^ Mean information processing speed (and standard deviation) based on the Digit Symbol Substitution test from the Wechsler Adult Intelligence Scale-Revised (Wechsler, 1981). ^c^ Mean vocabulary score (and standard deviation) based on the multiple choice section of the Mill Hill vocabulary test (Raven, Raven, & Court, 1988); maximum score = 33. ^d^ Mean digit span score (and standard deviation) based on the digit span test from the Wechsler Adult Intelligence Scale-Revised (Wechsler, 1981). ^e^ Older adults significantly different from young adults, *p* < .0001.
